# Study of the Chemical Composition and Biological Activity of the Essential Oil from Congona (*Peperomia inaequalifolia* Ruiz and Pav.)

**DOI:** 10.3390/plants12071504

**Published:** 2023-03-30

**Authors:** Eduardo Valarezo, Mercedes Herrera-García, Paola Astudillo-Dávila, Isabel Rosales-Demera, Ximena Jaramillo-Fierro, Luis Cartuche, Miguel Angel Meneses, Vladimir Morocho

**Affiliations:** Departamento de Química, Universidad Técnica Particular de Loja, Loja 110150, Ecuador

**Keywords:** biological activity, chemical composition, enantiomeric distribution, essential oil, *Peperomia inaequalifolia*

## Abstract

The species *Peperomia inaequalifolia*, commonly known as congona, is a succulent herbaceous plant belonging to the Piperaceae family, which is used for different purposes in traditional medicine. In this study, the chemical composition, enantiomeric distribution, and biological activity of essential oil isolated from the leaves of this species was determined. Hydrodistillation was used to isolate the essential oil. Gas chromatography coupled to mass spectrometry was used to determine the qualitative composition, a gas chromatograph equipped with a flame ionization detector was used to determine quantitative composition, and gas chromatography on an enantioselective column was used to determine enantiomeric distribution. Antibacterial activity was determined using the broth microdilution method, for which we used three Gram-positive cocci bacteria, a Gram-positive bacilli bacterium, and three Gram-negative bacilli bacteria. 2,2′-azinobis-3-ethylbenzothiazoline-6-sulfonic acid (ABTS) radical cations and 2,2-diphenyl-1-picrylhydryl (DPPH) radicals were used as reagents for determining the antioxidant activity of the essential oil. The spectrophotometric method was used to analyze the acetylcholinesterase inhibitory effect of the essential oil. The yield of leaves in essential oil was 0.16 ± 0.01% (*v*/*w*). Forty-three chemical compounds were identified in the essential oil, which represent 97.46% of the total composition. Sesquiterpene hydrocarbons were the most representative group, with 24 compounds (21.63%). The principal constituents were found to be elemicin (27.44 ± 1.35%), bisabolol <α-> (17.76 ± 1.38), myristicin (15.45 ± 0.86), methyl eugenol (6.22 ± 0.24), viridiflorene (6.81 ± 0.10), and safrole (6.68 ± 0.23). Three pairs of enantiomers were identified in the essential oil of *Peperomia inaequalifolia*. Essential oil presented a minimum inhibitory concentration (MIC) of 4000 μg/mL against *Enterococcus faecalis*, *Enterococcus faecium*, *Staphylococcus aureus*, *Listeria monocytogenes*, and *Escherichia coli*. The antioxidant activity of the essential oil was strong according to the DPPH and ABTS methods, with a half radical scavenging capacity (SC_50_) of 293.76 ± 3.12 µg/mL and 226.86 ± 0.05 µg/mL, respectively. Additionally, the essential oil reported moderate anticholinesterase activity, with an IC_50_ of 43.93 ± 1.05 µg/mL.

## 1. Introduction

The Piperaceae family comprises about 3700 species of herbaceous and woody plants worldwide. This family is widely distributed throughout the world, but is mostly found in tropical and subtropical regions [1,2]. In Ecuador, the Piperaceae family is representative of the flora of the country and around four genera have been recorded, including 441 species, of which 134 are endemic species. The species of this family are found in different Ecuadorian regions, but they are more common in the tropical and subtropical regions of the Amazon and the Andean regions [3].

The two most abundant genera of the Piperaceae family in Ecuador are *Piper* and *Peperomia*. The genus *Peperomia* Ruiz and Pav comprises 224 species, with 59 endemic species [3]. This genus is made up of perennial plants that grow in a wide variety of shapes and sizes and are native to tropical and subtropical regions around the world, including Central and South America, Africa, Asia, and Australia. Ecuador, being a country located in the tropical region of South America, is home to several species of *Peperomia*. Some common *Peperomia* species found in Ecuador include *P. galioides*, *P. macrostachya*, *P. pellucida*, *P. peltigera*, *P. rotundara*, *P. rotundifolia*, *P. serpens*, *P. urocarpa*, and *P. inaequalifolia*, among others [4]. Many of these species are used in traditional medicine to treat various ailments, including respiratory disorders, infections, and headaches. It has also been shown that some species, such as *Peperomia inaequalifolia*, in addition to its ornamental use, have important antibacterial and antifungal properties [5,6,7].

*Peperomia inaequalifolia* is a species native to South America and is found in countries such as Brazil, Peru, Ecuador, Colombia, and Venezuela. This plant is characterized by having small, round, dark green leaves with light veins. The leaves grow in a rosette shape and the plant can grow up to about 15 cm in height. The flowers are small and white or yellowish in color and grow in spikes. It is a succulent plant that can store water in its leaves, so it does not require frequent watering. It prefers well-drained, moist soils and warm, stable temperatures [8]. In Ecuador, *Peperomia inaequalifolia*, also known as *Peperomia congona*, is a native and cultivated herb. This species grows in the Andean region at an elevation of 1500–3500 m.a.s.l., mainly in the provinces of Azuay, Cañar, Carchi, and Chimborazo [4]. *Peperomia inaequalifolia* is commonly known as “congona”, “pataku yuyu”, “congonilla” and “cuncuna”. Regarding its uses and applications, the plant is used to prepare chicha and aromatic waters, and the leaves are used as a food additive (condiment) and for personal hygiene (shampoo) [9]. In ancient culture, this herb is used by shamans to treat the folk illness “mal aire” or “bad wind”. Other applications in traditional medicine include the use of the juice of the leaves and the liquid from the hot stem to treat earache and deafness, as well as the ingestion of an infusion of the leaves to treat heart conditions. This species is also used as a cardiac stimulant, to treat headaches, sterility, menstrual cramps, postpartum conditions, and kidney and liver conditions [3,4].

The chemical composition of fixed and volatile secondary metabolites from *Peperomia inaequalifolia* is not well known, and there are few studies available that report on the specific chemical compounds present in this species. However, some *Peperomia* species contain various compounds, including alkaloids, flavonoids, tannins, and organic acids [2]. For example, the leaves of *Peperomia pellucida*, a species related to *Peperomia inaequalifolia*, have been extensively investigated, demonstrating the presence of compounds such as ascorbic acid, carotenoids, chlorogenic acid, and quercetin, among others [10].

Some *Peperomia* species, including *P. inaequalifolia*, contain essential oils (EOs) in their leaves, stems, and roots. *Peperomia* essential oils are rich in compounds such as terpenes, aldehydes, esters, and alcohols, which give them a wide range of aromatic and medicinal properties [11]. These EOs have antibacterial, antifungal, anti-inflammatory, antispasmodic, and analgesic properties, and have been used in traditional medicine to treat various conditions (headache, insomnia, muscle pain, and arthritis) [12,13]. Additionally, *Peperomia* EOs are used in the perfumery and cosmetics industry [14]. However, to date no information has been found in the literature (indexed articles) on the chemical composition or biological activity of the essential oil (EO) of *Peperomia inaequalifolia*. This fact has motivated us to conduct this study with the aim of studying the chemical composition of the essential oil of *Peperomia inaequalifolia*, as well as its specific biological activity (antibacterial, antioxidant, and anticholinesterase) to determine its potential for medicinal use.

## 2. Results

### 2.1. Essential Oil Isolated

A total of 12,000 g (three distillations of 4000 g) of fresh (with a moisture of 82 ± 3% *w*/*w*) *Peperomia inaequalifolia* leaves (aerial part), were hydrodistilled in a Clevenger-type apparatus to isolate the essential oil. The amount of EO obtained was approximately 19 mL, which represents a yield in percentage (R%) of 0.16 ± 0.01% or 1.6 ± 0.1 mL/Kg.

### 2.2. Physical Properties of Essential Oil

The EO from *Peperomia inaequalifolia* was presented as an unctuous liquid with a sweet odor. Table 1 shows the mean values and standard deviations (SD) of the physical properties of essential oil. Generally, the essential oil of *Peperomia inaequalifolia* was a yellow liquid denser than water.

### 2.3. Chemical Composition of Essential Oil

The qualitative identification of the *Peperomia inaequalifolia* compounds was carried out by using gas chromatography coupled to mass spectrometry (GC–MS) and the quantification of their relative abundances was made by using gas chromatography equipped with the flame ionization detector (GC–FID). The compound numbers (CN) assigned according to their elution order, retention time (RT), calculated retention indices (RIC), reference retention indices (RIR), relative abundance (%), chemical formula (CF), and monoisotopic mass (MM) for each compound are shown in Table 2. Forty-three chemical compounds were identified in the EO of leaves from *Peperomia inaequalifolia*, which represent 97.46% of the total composition. The compounds were classified into four groups: monoterpene hydrocarbons (MH), oxygenated monoterpenes (OM), sesquiterpene hydrocarbons (SH), oxygenated sesquiterpene (OS), and other compounds (OC, non-terpenic compounds). In the number of compounds, SH was the most representative group with twenty-four compounds, followed by OS with twelve compounds. In terms of relative abundance, the most representative group was OC with 49.24%, and three of the six main compounds belonged to this group. The MH was the least representative group with 0.21%, and the presence of diterpenes was not determined. The principal constituents (>5%) are found to be OC elemicin (CN: 34, CF: C_12_H_16_O_3_, MM: 208.11 Da) with 27.44 ± 1.35%, myristicin (CN: 32, CF: C_11_H_12_O_3_, MM: 192.08 Da) with 15.45 ± 0.86 and methyl eugenol (CN: 11, CF: C_11_H_14_O_2_, MM: 178.10 Da) with 6.22 ± 0.24, OS bisabolol <α-> (CN: 43, CF: C_15_H_26_O, MM: 222.20) with 17.76 ± 1.38 and viridiflorene (6.81 ± 0.10), and OM safrole with 6.68 ± 0.23.

### 2.4. Enantiomeric Analysis

Using a column with an enantioselective stationary phase, it was possible to separate three pairs of enantiomers from the EO of *Peperomia inaequalifolia* leaves. Table 3 shows the retention time (RT), enantiomers, retention indices (RI), enantiomeric distribution (ED), and enantiomeric excess (e.e.), for each pair of compounds. The (+)-β-pinene was found to be pure with 100%. In addition, a racemic mixture was identified, with ED close to 50%, for the enantiomers of β-bisabolene, although it was impossible to establish the order of elution between the enantiomers (+) and (−).

### 2.5. Antimicrobial Activity

The microdilution broth method was used to determine the antibacterial activities of the EO of leaves from *Peperomia inaequalifolia*. Table 4 shows the tested microorganisms and minimum inhibitory concentration (MIC) values of EO and positive control, in addition to the values of the negative control. Ampicillin was used as a positive control for *Enterococcus faecalis*, *Enterococcus faecium*, and *Staphylococcus aureus*, and ciprofloxacin for *Listeria monocytogenes*, *Escherichia coli*, *Pseudomonas aeruginosa*, and *Salmonella enterica*. Dimethylsulfoxide at 5% was used as a negative control. The *Peperomia inaequalifolia* EO reported MIC values of 4000 µg/mL against Gram-positive cocci, Gram-positive bacilli, and *Escherichia coli* (Gram-negative bacilli).

### 2.6. Antioxidant Activity

The antioxidant activity of essential oil from *Peperomia inaequalifolia* was determined using the DPPH and ABTS methods. The DPPH method is based on the scavenging capacity of the essential oil against the radical 2,2-diphenyl-1-picrylhydrazyl radical (DPPH^•^), and in the ABTS method scavenging capacity was determined against the radical ion 2,2′-azino-bis(3-ethylbenzothiazoline-6-sulfonic acid) radical cation (ABTS^•+^). Table 5 shows the scavenging capacity (SC_50_) in µg/mL of the essential oil and the positive control (trolox). The maximum evaluated concentration was 1000 µg/mL.

### 2.7. Anticholinesterase Activity

**Figure 1:**
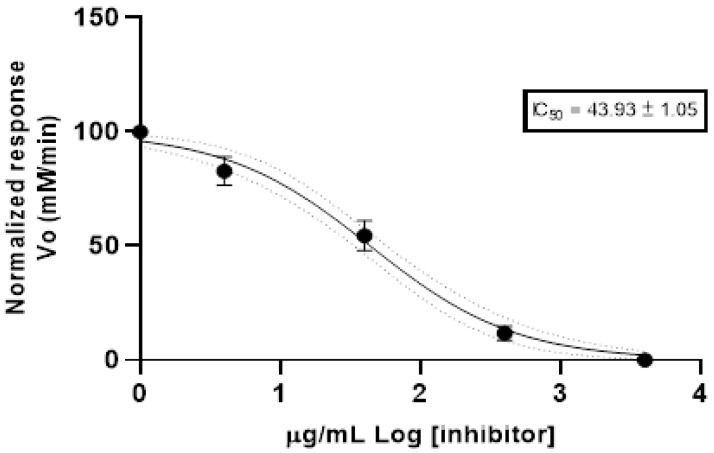
Anticholinesterase activity of essential oil from *Peperomia inaequalifolia*.

The anticholinesterase (anti-AChE) activity was determined using the spectrophotometric method. Figure 1 shows the Log of concentration of EO and normalized response rate of reaction of acetylcholinesterase. The results were reported as half maximal inhibitory concentration (IC_50_) value. The *Peperomia inaequalifolia* EO reported an IC_50_ value of 43.93 ± 1.05 µg/mL. The positive control (donepezil) exhibited an IC_50_ value of 12.40 ± 1.35 µg/mL.

## 3. Discussion

The extraction yield of essential oil of *P. inaequalifolia* showed a value of 0.16 ± 0.01%. The oil was yellow in appearance with a refractive index of 1.6714 ± 0.0034. Noriega et al. in 2015 [15] (non-indexed publication) reported a similar yield value for *P. inaequialifoila*.

The essential oils are secondary metabolites from plants and the quantity varies depending on the part of the plant, e.g., leaves, roots, branches, fruits, and bark. It is known that the extraction yield depends on the extraction procedure, processing factors, cultivar practices, and postharvest processing. Generally, the yield of essential oils has been reported between 0.01% and 3% [16], according to the categorization of extraction yield for essential oils cited by Molares et al. In 2009 [17], the yield for the EO of *P. inaequalifolia* was reported to be low with a value of less than 5 mL/kg.

The main volatile compounds identified in the EO of *P. inaequialifolia* were elemicin 27.44 ± 1.35%, bisabolol <α-> 17.76 ± 1.38%, myristicin 15.45 ± 0.86%, viridiflorene 6.81 ± 0.10%, safrole 6.68 ± 0.23% and methyl eugenol 6.22 ± 0.24%. The majority of identified compounds (49.24%) were grouped as OC (non-terpenoid compounds), while sesquiterpene compounds (21.63%) were quite similar to oxygenated sesquiterpenes (19.53%). These results can be compared with the chemical composition reported by Noriega et al. (2015) [15], who identified 14 compounds representing 93% of volatile compounds with the main compounds (>5%) being safrole (32.10%), 11-αH-himachal-4-en-1-β-ol (25.29%), myristicin (13.29%), elemicin (10.07%), and viridiflorol (5.24%). Although there are differences in abundance, the same compounds are present except for 11-αH-himachal-4-en-1-β-ol. Safrole was present as a characteristic compound in the species of the Piperaceae family as reported by Barbosa et al. in 2012 [18]. Ponce Cobos and Castro in 2017 studied the essential oil of *P. galioides* Kunth and reported the main components as 1,3-benzodioxole, 4-methoxy-6-(2-propenyl)-(71.2%), and safrole (10.34%) [19]. On the other hand, Miranda et al. (2021) evaluated the seasonal variations in the chemical composition of the essential oil of *P. circinnata*; however, safrole was not identified [20]. The US EPA (United States Environmental Protection Agency) has evaluated the carcinogenicity of safrole and classifies it as possibly carcinogenic to humans (Group B2: the agent (mixture) is possibly carcinogenic to humans). Hence, the use of substances containing safrole is regulated by legal restriction in USA, and safrole has been prohibited in soft drinks since the 1970s. In Europe, a maximum concentration of 1-ppm safrole in food and beverages is allowed. The international Fragrance Association (IFRA) recommends that essential oils containing safrole should not be used at levels in which the total concentration exceeds 0.01% in consumer products. Safrole is allowed in China (State Food and Drug Administration of China, SFDA of China) in concentrations below 1 mg/L [21]. Moreover, elemicin and myristicin have been reported as potential toxins in foods [22].

The enantiomeric analysis shows that pure (+)-β-pinene was present (Table 3), and two racemic mixtures were identified for (+/−)-α-muurolene (e.e 47.75%) and (+/−)-β-bisabolene (e.e 22.42%). To the best of our knowledge, this is the first report of enantioselectivity analysis for the EO of *P. inaequialifolia*. The identification of enantiomers in essential oils is important to determine the bioactivity, since enantiomers of a compound have different biological activities [23]. The enantiomeric distribution of an EO helps to determine its quality and purity, due to the fact that the percentage of each enantiomer is unique to a specific EO. Therefore, the enantiomeric analysis allows us to consider the potential application of EO [23,24]; furthermore, enantiomeric compounds could affect the sensorial properties [25].

The antimicrobial activity was analyzed. The results, presented in Table 4, show MIC values of 4000 µg/mL and >4000 µg/mL. According to Van Vuuren and Holl in 2017, MIC values for essential oils higher than 1001 µg/mL are considered to be inactive in antimicrobial tests [26]. Noriega et al. in 2015 evaluated the antimicrobial activity of the EO of *P. inaequialifolia* using the disk diffusion method and reported strong activity of MIC 100 µg/mL for Gram-positive bacteria *Staphylococcus aureus* subsp. *aureus* and *Streptococcus mutans*, however, these values could not be directly compared due to the different antimicrobial methods [15]. There are few articles in the literature presenting the antimicrobial activity of *Peperomia* species. Okoh et al. in 2017 evaluated the EO of *P. pellucida* (L.) Kunth against seven strains of bacteria with MIC of 150 µg/mL for Listeria *ivanovii*, an MIC value of 200 µg/mL for *S. aureus*, *Enterobacter cloacae*, *Escherichia coli*, *Vibrio parahaemolyticus*, *Mycobacterium smegmatis*, and *Streptococcus uberis*. However, it is necessary to mention that the main components are different [27].

The possible application of essential oils is related to their antioxidant capacity. The EO of *P. inaequilifolia* showed weak antioxidant capacity according to the DPPH and ABTS assays. However, Noriega et al., 2015 reported an SC_50_ value of 2220 µg/mL for the DPPH assay [15]. The variation in the chemical composition explains the difference in antioxidant capacity. Okoh et al. in 2017 reported the SC_50_ 1670 µg/mL and 1940 µg/mL for the DPPH and ABTS methods, respectively, for EO from *Peperomia pellucida* [27]. Generally, the antioxidant activity corresponds to the main components of the essential oils. Elemicin is an alkenylbenzene reported with pharmacological effects as antioxidant by Surveswaran et al. in 2006 [28] and the SC_50_ for DPPH was minor to 100 µg/mL according to Al-Qahtani et al., 2022 [29]. On the other hand, α-bisabolol has also has been reported to have pharmacological effects, including in vivo antioxidant capacity [30].

Regarding anticholinesterase activity, this is the first report for the EO of *P. inaequalifolia* with an IC_50_ of 43.93 ± 1.05 µg/mL, which could be considered moderate potency according to the classification of anti-AChE proposed by Santos et al. in 2018 who proposed three categories: high potency IC_50_ < 20 µg/mL; moderate potency 20 < IC_50_ < 200 µg/mL; and low potency 200 < IC_50_ < 1000 [31]. The value is quite similar to that reported for *A. cherimola* EO with an IC_50_ of 41.51 µg/mL [32]. The results of anticholinesterase activity are relevant in researching treatments for Alzheimer disease, and Benny and Tomas in 2019 reported the neuroprotective effect of EO and the pure compounds [33].

## 4. Materials and Methods

### 4.1. Materials

Helium was purchased from INDURA (Quito, Ecuador). Mueller Himton broth, Mueller Hinton II broth, and fluid thioglycollate medium were purchased from DIPCO (Quito, Ecuador). The standard aliphatic hydrocarbons were purchased from ChemService (West Chester, PA, USA). Acetylcholinesterase (AChE), acetylthiocholine (AcSCh), dichloromethane (DMC), dimethyl sulfoxide (DMSO), methanol (MeOH), 2,2-diphenyl-1-picrylhydryl (DPPH), 2,2′-azinobis-3-ethylbenzothiazoline-6-sulfonic acid (ABTS), 5,5′-dithiobis (2-nitrobenzoic acid) (DTNB), butylated hydroxytoluene (BHT), donepezil, magnesium chloride hexahydrate, phosphate-buffered saline (PBS), sodium sulfate anhydrous, trolox, and tris hydrochloride (Tris-HCl) were purchased from Sigma-Aldrich (San Luis, MO, USA). All chemicals were of analytical grade and used without further purification.

### 4.2. Plant Material

The leaves of *Peperomia inaequalifolia* were collected in the surroundings of the Guayllabamba Parish, Quito Canton, Pichincha Province. The collection was carried out in a valley that is located at 0°04′45″ south longitude and 78°21′06″ west latitude and an altitude of 2171 m a.s.l. After being collected, the plant material was stored and transferred in airtight plastic containers. The environmental conditions in the collection and transfer were a pressure of 79 KPa and a temperature of 18–20 °C. The botanical identification of the specimen was performed by Dr. Vladimir Morocho. A voucher specimen was preserved in the Herbarium of the Universidad Técnica Particular de Loja (HUTPL). The method AOAC 930.04-1930, loss on drying (moisture) in plants, was used to determine the moisture of plant material.

### 4.3. Essential Oil Isolation

A Clevenger-type apparatus was used for isolating essential oil, in which the extraction of the oil was carried out by hydrodistillation, for which an 80 L distiller was used, in which approximately 18 L of water was placed. The process was maintained for 3 h, counted from the fall of the first drop of distillate. The condensed essential oil was separated from the water by decantation, because the EO turned out to be denser than the water, and the separation of the water–oil mixture was carried out in a special decanter. Then, the essential oil was dried using anhydrous sodium sulfate and stored at 4 °C in amber sealed vials until being used in analysis. Yield in percentage was calculated as R% = (volume of EO in mL/weight of plant material in g)∗100.

### 4.4. Identification and Quantification of Essential Oil Compounds

The analysis of chemical composition was carried out in a gas chromatograph (GC) (model 6890N series, Agilent Technologies, Santa Clara, CA, USA). For qualitative analysis, the GC was coupled to a quadrupole mass spectrometer (MS) (model Agilent series 5973 inert, Agilent Technologies, Santa Clara, CA, USA) and for quantitative analysis the GC was equipped with a flame ionization detector (FID). In both cases, a nonpolar chromatographic column (Agilent J&W DB-5ms Ultra Inert GC column, Agilent Technologies, Santa Clara, CA, USA) with stationary phase 5%-phenyl-methylpolyxilosane, 30 m long, 0.25 mm of internal diameter and 0.25 µm of stationary phase thickness was used. The GC was equipped with a split/splitless autosampler (model 7683, Agilent Technologies, Santa Clara, CA, USA). The supply of hydrogen for the FID was carried out using a gas generator (model 9150, Packard, Conroe, TX, USA). The EO sample was prepared at 1% (*v*/*v*) by putting 10 μL of EO and 990 μL dichloromethane in an amber vial. For the qualitative and quantitative analyses, a 1 μL sample was injected in the split mode with a partition ratio of 40:1, at a temperature of 220 °C and a pressure of 11 psi. In both cases, the chromatographic run began maintaining the initial temperature of 50 °C for 3 min, then the temperature was increased by 3 °C/min until reaching the final temperature of 230 °C, which was maintained for 3 min. For GC–MS, a constant flow of helium was maintained at a rate of 0.9 mL/min and a velocity of 23 cm/s, and for GC–FID the flow was 1.0 mL/min and the speed was 40 cm/s. For the identification of the chemical compounds of the essential oil, two complementary methods were used: the mass spectra and the retention index (RI). The mass spectrum of the compound was compared with the mass spectra from the equipment library, which gives us the qualitative information of the compound. The RI of each compound was calculated with Equation (1) [34] and the obtained value was compared with the RI reported in the book of Adams, R.P. [35] and in the online database NIST Chemistry WebBook [36]. For a compound to be considered identified, it must be identified by both methods.

(1) $$\mathrm{RI}=100C+100\frac{\left(\mathrm{RTx}-\mathrm{RTn}\right)}{\left(\mathrm{RTN}-\mathrm{RTn}\right)}$$

where, C is the carbon number of aliphatic hydrocarbons (C_9_ to C_25_) that elutes before the compound of interest, RTx is the retention time of the compound of interest, RTn is the retention time of aliphatic hydrocarbons that elutes the compound of interest, and RTN is the retention time of hydrocarbons that elutes after the compound of interest.

### 4.5. Enantioselective Analysis

The determination of the enantiomeric distribution of an EO is a key aspect of the analysis of its chemical composition. The importance of this analysis is evident if we consider that two enantiomers, chemically indistinguishable in a non-chiral chemical analysis, show different biological properties. The enantiomers can present different olfactory properties. For this reason, two EOs which present a similar chemical composition can be characterized by two different aromas [32]. This phenomenon cannot be explained by classical chemical analysis but can be understood by comparing the enantioselective profiles. For enantiomeric analysis, gas chromatography (Trace 1310, Thermo Fisher Scientific, Waltham, MA, USA) coupled to a mass spectrometer (quadrupole) (ISQ 7000, Thermo Fisher Scientific, Waltham, MA, USA) was used. Analyses were performed on an enantioselective GC column (MEGA-DEX DMT-Beta, Mega, Legnano, MI, Italy) with 30 m length, 0.25 m internal diameter, and 0.25 μm thick stationary phase (2,3-diethyl-6-tert-butyldimethylsilyl-β-cyclodextrin). Sample preparation, amount injected, injection temperature, and partition radius were those described for GC–MS. The carrier gas used was helium, with a flow of 1.0 mL/min and a speed of 40 cm/s. The chromatographic run began by maintaining the oven at 60 °C for 5 min, then the temperature was increased with a ramp of 2 °C/min up to 230 °C, finally this temperature was maintained for 5 min. The calculation of the enantiomeric excess and elution order was carried out according to the procedures previously described by Morocho et al. [37].

### 4.6. Antimicrobial Activity

The antibacterial activity of the essential oil was tested against seven strains of bacteria, three Gram-positive cocci bacteria: *Enterococcus faecalis* (ATCC 19433), *Enterococcus faecium* (ATCC 27270) and *Staphylococcus aureus* (ATCC 25923); a Gram-positive bacilli bacterium: *Listeria monocytogenes* ATCC 19115; and three Gram-negative bacilli bacteria: *Escherichia coli* O157:H7 (ATCC 43888), *Pseudomonas aeruginosa* (ATCC 10145), and *Salmonella enterica* subs enterica serovar Thypimurium WDCM 00031, derived (ATCC 14028). The broth microdilution method was used to determine this activity, and the procedures were performed as previously described by Valarezo et al. [38]. The maximum evaluated concentration was 4000 µg/mL. Ampicillin and ciprofloxacin were used as a positive control and DMSO was used as a negative control.

### 4.7. Evaluation of Antioxidant Capacity

The DPPH and ABTS methods were used to determine the free radical scavenging activity of EO from *Peperomia inaequalifolia*. The antioxidant capacity of EO was determined according to the procedure described by Salinas et al. [39], using a UV spectrophotometer (Genesys 10S UV-Vis Spectrophotometer, Thermo Fisher Scientific, Waltham, MA, USA). In the DPPH method, a 2,2-diphenyl-1-picrylhydrazyl radical (DPPH^•^) was produced from the reagent 2,2-diphenyl-1-picrylhydrazyl (DPPH), and the absorbance of the samples was measured at a wavelength of 515 nm. In the ABTS method, a 2,2′-azinobis(3-ethylbenzothiazoline-6-sulfonic acid) radical cation (ABTS^•+^) was produced from reagent 2,2′-azinobis (3-ethylbenzothiazoline-6-sulfonic acid) (ABTS) and the measurement of the absorbance of the samples was carried out at a wavelength of 734 nm. The SC50, which is the concentration value necessary for the EO to have half radical scavenging capacity, was used to express the antioxidant activity. Trolox and methanol were used as positive and negative controls, respectively.

### 4.8. Anticholinesterase Activity

Cholinesterase is a term referring to the two enzymes acetylcholinesterase and pseudocholinesterase. Both compounds catalyze the hydrolysis of excess neurotransmitter acetylcholine, and the excessive activation caused by acetylcholine would produce damage to the neuron or muscle. A cholinesterase inhibitor is known as an anticholinesterase compound [37]. Anticholinesterase compounds are also used for treating myasthenia gravis, glaucoma, and Alzheimer’s disease. Synthetic anticholinesterase compounds are potent neurotoxins of moderate effectiveness, high cost, and short half-life that can also cause gastrointestinal disturbances. For this reason, compounds isolated from natural products (plants) such as EOs are currently increasingly being explored for their anticholinesterase properties and their better secondary effects [31]. The spectrophotometric method was used to determine the acetylcholinesterase inhibitory effect of the EO of leaves from *Peperomia inaequalifolia*. Acetylthiocholine was used as the substrate to detect the inhibition of AChE as previously described by Valarezo et al. [21]. The tested sample solutions from the EO were made by dissolving 10 mg in 1 mL MeOH, then, the reaction mixture containing 40 µL of Buffer C, 20 µL of the tested sample solution, 20 µL of AcSCh, and 100 µL of DTNB was preincubated for 3 min at 25 °C. The enzymatic reaction was started with the addition of 20 µL of 0.5 U/mL AChE and then incubated at 25 °C for 30 min. The amount of product released was monitored in a microplate spectrophotometer (EPOCH 2, BioTek, Winoo-ski, VT, USA) every 1 min, at 405 nm. The IC50 was used to express the anticholinesterase activity. IC_50_ is the concentration of EO required for 50% inhibition. Methanol and donepezil hydrochloride were used as negative and positive controls, respectively.

### 4.9. Statistical Analysis

All procedures were performed in triplicate, except the identification of essential oil compounds, enantioselective analysis, and antimicrobial activity, which were performed nine times. The data were collected in a Microsoft Excel sheet. The statistical software Minitab 17 (Version 17.1.0., Minitab LLC., State College, PA, USA) was used to calculate the measures of central tendency and standard deviation.

## 5. Conclusions

The enantiomeric distribution, antimicrobial activity, antioxidant capacity, and anticholinesterase activity of essential oil from the leaves of *Peperomia inaequalifolia* were determined for the first time. Forty-three chemical compounds and three pairs of enantiomers were identified in the essential oil. The main compound was elemicin. Essential oil presented a strong antioxidant activity and moderate anticholinesterase activity. With this research, new information is provided on the species of aromatic plants of Ecuador, thus contributing to the knowledge of Ecuadorian biodiversity. It was determined that safrole is present in essential oil from *Peperomia inaequalifolia*. The use of natural substances containing safrole or products formulated with it is regulated by international committees (US EPA, IFRA, SFDA of China, etc.). Furthermore, elemicin and myristicin have also been reported as potential toxins in foods. Therefore, it is recommended that this essential oil is not used in applications related to human use or consumption.

## Figures and Tables

**Table 1:** Physical properties of the essential oil of *Peperomia inaequalifolia*.

	*Peperomia inaequalifolia* EO	
	Mean	SD
Density, (g/cm^3^)	1.0232	0.0006
Refractive index, *n*^20^	1.6714	0.0034
Specific rotation, [α] (°)	+16.21	0.25
Subjective color	Yellow	
RGB color values	R:250, G:250, B:40	
CMYK color values	C:0, M:0, Y:84, K:2	

**Table 2:** Chemical composition of essential oil from the leaves of *Peperomia inaequalifolia*.

CN	RT	Compound	RIC	RIR	%	SD	Type	CF	MM (Da)
1	9.16	Sabinene	969	969	tr	-	MH	C_10_H_16_	136.13
2	9.69	Pinene <β->	974	974	0.05	0.01	MH	C_10_H_16_	136.13
3	10.35	Ethyl hexanoate	997	997	0.13	0.01	OC	C_8_H_16_O_2_	144.12
4	11.9	Sylvestrene	1025	1025	0.16	0.01	MH	C_10_H_16_	136.13
5	12.15	Cineole <1,8->	1028	1026	0.17	0.01	OM	C_10_H_18_O	154.14
6	28.82	Safrole	1283	1285	6.68	0.23	OM	C_10_H_10_O_2_	162.07
7	31.36	Cubebene <α->	1347	1348	0.47	0.04	SH	C_15_H_24_	204.19
8	32.85	Copaene <α->	1372	1374	0.16	0.02	SH	C_15_H_24_	204.19
9	34.22	Chamipinene <α->	1401	1396	0.47	0.04	SH	C_15_H_24_	204.19
10	34.39	Sibirene	1403	1400	0.29	0.03	SH	C_15_H_24_	204.19
11	34.76	Methyl eugenol	1406	1403	6.22	0.24	OC	C_11_H_14_O_2_	178.10
12	34.88	Cedrene <α->	1413	1410	0.32	0.02	SH	C_15_H_24_	204.19
13	35.03	Caryophyllene <(E)->	1418	1417	0.25	0.07	SH	C_15_H_24_	204.19
14	35.55	Copaene<β->	1430	1430	0.21	0.05	SH	C_15_H_24_	204.19
15	35.93	Aromadendrene	1438	1439	0.62	0.08	SH	C_15_H_24_	204.19
16	36.46	Himachalene <α->	1450	1449	0.31	0.04	SH	C_15_H_24_	204.19
17	36.67	Farnesene <(E)-β->	1454	1454	3.06	0.07	SH	C_15_H_24_	204.19
18	36.75	Prenyl limonene <trans->	1456	1457	0.29	0.05	SH	C_15_H_24_	204.19
19	36.94	Aromadendrene <allo->	1460	1458	0.26	0.02	SH	C_15_H_24_	204.19
20	37.15	Acoradiene <α->	1465	1464	0.16	0.01	SH	C_15_H_24_	204.19
21	37.52	Cadina-1(6),4-diene <trans->	1474	1475	1.14	0.03	SH	C_15_H_24_	204.19
22	37.68	Muurolene <γ->	1478	1478	1.18	0.02	SH	C_15_H_24_	204.19
23	37.81	Amorphane <cis-4,10-epoxy->	1481	1481	1.02	0.04	OS	C_15_H_26_O	222.20
24	37.94	Amorphene <α->	1484	1483	0.16	0.01	SH	C_15_H_24_	204.19
25	38.38	Viridiflorene	1493	1496	6.81	0.10	SH	C_15_H_24_	204.19
26	38.60	Bicyclogermacrene	1498	1500	2.23	0.12	SH	C_15_H_24_	204.19
27	38.76	Muurolene <α->	1502	1500	1.16	0.02	SH	C_15_H_24_	204.19
28	38.99	Farnesene <(E,E)-α->	1507	1505	0.23	0.01	SH	C_15_H_24_	204.19
29	39.13	Bisabolene <(Z)-α->	1508	1506	0.22	0.01	SH	C_15_H_24_	204.19
30	39.39	Amorphene <δ->	1513	1511	0.33	0.03	SH	C_15_H_24_	204.19
31	39.60	Cadinene <γ->	1515	1513	1.25	0.02	SH	C_15_H_24_	204.19
32	40.21	Myristicin	1519	1517	15.45	0.86	OC	C_11_H_12_O_3_	192.08
33	40.41	Cadinene <α->	1537	1537	0.05	0.00	SH	C_15_H_24_	204.19
34	41.33	Elemicin	1556	1555	27.44	1.35	OC	C_12_H_16_O_3_	208.11
35	41.48	Nerolidol <(E)->	1559	1561	0.67	0.02	OS	C_15_H_26_O	222.20
36	42.21	Spathulenol	1575	1577	0.32	0.06	OS	C_15_H_24_O	220.18
37	42.54	Globulol	1587	1590	0.12	0.01	OS	C_15_H_26_O	222.20
38	42.87	Viridiflorol	1589	1592	0.06	0.01	OS	C_15_H_26_O	222.20
39	43.46	Cedrol	1602	1600	0.13	0.01	OS	C_15_H_26_O	222.20
40	44.06	Cubenol <1,10-di-epi->	1615	1618	0.19	0.01	OS	C_15_H_26_O	222.20
41	44.75	Muurolol <α-> (=Torreyol)	1640	1644	0.17	0.02	OS	C_15_H_26_O	222.20
42	45.43	Caryophyllene <14-hydroxy-(Z)->	1660	1666	0.11	0.01	OS	C_15_H_24_O	220.18
43	46.04	Bisabolol <α->	1688	1685	17.76	1.38	OS	C_15_H_26_O	222.20
		Monoterpene hydrocarbons			0.21				
		Oxygenated monoterpenes			6.85				
		Sesquiterpene hydrocarbons			21.63				
		Oxygenated sesquiterpene			19.53				
		Other compounds			49.24				
		Total identified			97.46				

CN: compound number: SD: standard deviation; Tr: traces.

**Table 3:** Chiral compounds present in the essential oil of the leaves from *Peperomia inaequalifolia*.

RT	Enantiomers	RI	ED (%)	e.e (%)
8.53	(+)-β-Pinene	992	100	100
35.44	(+/−)-α-Muurolene	1521	73.87	47.75
35.71		1526	26.13	
36.41	(+/−)-β-Bisabolene	1538	38.79	22.42
36.68		1543	61.21	

**Table 4:** Antibacterial activity of essential oil from *Peperomia inaequalifolia*.

Microorganism	Essential Oil	Positive Control	Negative Control
	MIC (µg/mL)		
Gram-positive cocci			
*Enterococcus faecalis* (ATCC 19433)	4000	0.78	+
*Enterococcus faecium* (ATCC 27270)	4000	0.39	+
*Staphylococcus aureus* (ATCC 25923)	4000	0.39	+
Gram-positive bacilli			
*Listeria monocytogenes* (ATCC 19115)	4000	1.56	+
Gram-negative bacilli			
*Escherichia coli* O157:H7 (ATCC 43888)	4000	1.56	+
*Pseudomonas aeruginosa* (ATCC 10145)	>4000	0.39	+
*Salmonella enterica* subs enterica serovar Thypimurium WDCM 00031, derived (ATCC 14028)	>4000	0.39	+

+: normal growth.

**Table 5:** Antioxidant activity of essential oil from *Peperomia inaequalifolia*.

Sample	DPPH	ABTS
	SC_50_ (µg/mL) ± SD	
*Peperomia inaequalifolia* oil	293.76 ± 3.12	226.86 ± 0.05
Trolox	29.99 ± 1.1	23.27 ± 1.1

## Data Availability

Data are available from the authors upon reasonable request.
